# Heterologous Expression and Antimicrobial Mechanism of a Cysteine-Rich Peptide from Barnacle *Pollicipes pollicipes*

**DOI:** 10.3390/microorganisms13061381

**Published:** 2025-06-13

**Authors:** Zhicheng He, Zixun Fei, Huishao Shi, Meichuan Huang, Liumi Wei, Junjian Wang, Peng He, Wei Zhang

**Affiliations:** 1 College of Marine Science BGU, Beibu Gulf University, Qinzhou 535011, China; hzc17740977790@163.com (Z.H.); fzx3431504249@163.com (Z.F.); shuiskx@163.com (H.S.); hmc204704@163.com (M.H.); wlmbbgu@163.com (L.W.); wangjunjian@bbgu.edu.cn (J.W.); 2 Guangxi Key Laboratory of Marine Environmental Change and Disaster in Beibu Gulf, Beibu Gulf University, Qinzhou 535011, China

**Keywords:** antimicrobial peptides, cysteine-rich, antibacterial mechanism, *Pollicipes pollicipes*, antibiotic

## Abstract

The escalating crisis of antimicrobial resistance in aquaculture, driven by the indiscriminate use of antibiotics, underscores the urgent need to develop novel anti-infective agents. This study addresses this requirement by investigating cysteine-rich antimicrobial peptides (AMPs) in understudied crustacean species. A cysteine-rich AMP, designated *Pp*Rcys1, was identified and characterized from the genome of *Pollicipes pollicipes*. *Pp*Rcys1 comprises 104 amino acids, with 85 residues forming the mature peptide region, and exhibits random coils, a CSαβ-fold, and one β-sheet. Our findings demonstrated that recombinant *Pp*Rcys1 (r*Pp*Rcys1) possesses broad-spectrum antimicrobial activity against three Gram-positive bacteria (*Staphylococcus aureus*, *Bacillus* sp. T2, and *Streptococcus agalactiae*) and four Gram-negative bacteria (*Aeromonas hydrophila*, *Escherichia coli*, *Vibrio alginolyticus*, and *Acinetobacter* sp. L3), with minimum inhibitory concentrations ranging from 8 to 32 μM. It exerts antimicrobial effects by inducing membrane disruption without impacting bacterial protease activity, DNA migration, or respiratory chain reductase activity. Further investigation is warranted to determine whether it can target and interfere with intracellular bacterial processes. Our discovery and characterization of this novel AMP provide a promising foundation for its development as an alternative to antibiotics.

## 1. Introduction

In aquaculture, antibiotics play a critical role in controlling disease outbreaks and enhancing production. However, their extensive use has also resulted in significant adverse consequences [1]. The presence of antibiotics at subtherapeutic concentrations in farming environments imposes strong selective pressure on bacteria, promoting resistance acquisition through gene mutations, plasmid transfers, and other mechanisms. This has led to a growing population of drug-resistant bacteria, which threatens the sustainable development of the aquaculture industry. Therefore, limiting antibiotic use and investigating alternative therapeutic strategies are imperative to address this challenge [2]. Antimicrobial peptides (AMPs), naturally occurring molecules with broad-spectrum activity against diverse microorganisms, represent promising candidates for the development of new therapeutic strategies [3,4].

AMPs are integral components of the innate immune response, functioning against both bacterial and fungal infections [5,6]. Among the vast array of AMPs identified, more than half have been characterized in arthropods, which include both crustaceans and insects. Since the initial discovery of a crustacean AMP in *Carcinus maenas* in 1996 [7], only 73 crustacean AMPs have been cataloged in the Antimicrobial Peptide Database 3 (APD3, http://aps.unmc.edu/AP/, accessed on 12 February 2025) [8]. Given the substantial biodiversity of crustaceans, there remains considerable potential for identifying additional AMPs in this group.

Cysteine-rich AMPs constitute one of the most diverse and widely distributed peptide families, exhibiting notable structural diversity and a broad spectrum of antimicrobial activities [9,10,11]. Notable examples include defensins, charybdotoxins, tachyplesins, and crustin [10,12,13]. Representative subgroups of cysteine-rich peptides include those containing with the CSαβ motif and those forming hairpin-like β-sheet structure [10,11]. These peptides commonly disrupt microbial cytoplasmic membranes [10] or penetrate cells and interfere with intracellular functions such as protein synthesis and DNA replication [14,15].

*Pollicipes pollicipes* (Crustacea: Cirripedia) is a barnacle species commonly found on sea reefs [16]. In the same sea area, barnacles are frequently exposed to higher levels of pathogenic microorganisms than shellfish, yet there are no reported cases of pathogen-induced mortality in barnacles [17,18,19]. In addition, barnacles exhibit the ability to eliminate biofilms from their surroundings [20]. Based on these observations, we hypothesized that barnacles produce antimicrobial peptides (AMPs) with potent antimicrobial properties.

To address the critical need for new antimicrobial agents in aquaculture, we established a comprehensive discovery pipeline for cysteine-rich AMPs and identified a promising candidate, *Pp*Rcys1. Its antimicrobial spectrum and mechanism of action were systematically investigated using computational and molecular biology. Preliminary toxicity evaluations were also conducted to assess its safety profile.

## 2. Materials and Methods

### 2.1. Bacterial Strains and Culture Conditions

The experimental bacteria included three Gram-positive strains: *Staphylococcus aureus* (ATCC 6538), *Bacillus* sp. T2, and *Streptococcus agalactiae* (ATCC13813), and four Gram-negative strains: *Aeromonas hydrophila* (ATCC35654), *Escherichia coli* (ATCC25922), *Vibrio alginolyticus* (ATCC17749), and *Acinetobacter* sp. L32. These strains, originally preserved as glycerol stocks at −80 °C, were kindly provided by Professor Xiaohui Cai [21] and Professor Chaogang Wang [22]. Before the experiment began, bacteria were inoculated into 2 mL of medium and cultured at 37 °C with shaking at 200 rpm for 12 h to activate them. Specifically, *S. aureus*, *S. agalactiae*, *E. coli*, *Acinetobacter* L32, and *A. hydrophila* were cultured in Luria–Bertani (LB) medium (ST163, Beyotime, Shanghai, China), whereas *V. alginolyticus* was cultured in Zobell Marine Broth 2216 (2216E) medium (HB0132, Haibo, Qingdao, Shandong, China).

### 2.2. Prediction and Identification of Cysteine-Rich AMPs

The genome information for *P. pollicipes* was obtained from the NCBI database (NCBI accession number: GCF_011947565.3). Cysteine-rich AMPs typically consist of approximately 100 amino acids and contain signal peptides, which are located in the extracellular space [23,24]. Cysteine-rich peptides, such as Mersacidin, Laterosporulin, Subtilosin A, Thuricin CDα, Thuricin CDβ, and Thuricin H, exhibit significant antibacterial activity and generally contain more than 10% cysteine content [25]. Using a custom program developed by our team, we selected sequences with fewer than 105 amino acids, uncharacterized proteins according to the NCBI BLAST database, and those with a cysteine content exceeding 10% (see the Supplementary Code S1). SignalP 6.0 was employed to predict the presence of signal peptides [26], whereas WoLF PSORT was used to determine subcellular localization, specifically targeting extracellular sequences [27]. The sequences were ranked based on cysteine content, with the highest-ranking sequence designated as *Pp*Rcys1. AMP prediction was performed using the prediction function of the APD3 database. The 3D structure of *Pp*Rcys1 was predicted using AlphaFold2 [28]. Additionally, the physicochemical properties of the protein were predicted using ProtParam (accessed on 1 February 2025; https://web.expasy.org/protparam/).

### 2.3. Expression and Purification of Recombinant PpRcys1 (rPpRcys1)

Recombinant *Pp*Rcys1 was produced using previously reported protocols [18]. Briefly, the coding sequences for the mature peptide regions (see Supplementary Table S1) were condon-optimized for *E. coli*, with BamHI and XhoI restriction sites appended to both ends. These sequences were chemically synthesized by General Biosystems (Chuzhou, China) and inserted into the pSmartI vector (containing a His-SUMO tag) via BamHI and XhoI restriction sites. The resulting recombinant plasmid, pSmartI-*Pp*Rcys1 (see Supplementary Figure S1), was 5814 bp in size. Transformation into *E. coli* BL21 (DE3) was performed using the heat shock method, and transformants were selected on LB agar supplemented with kanamycin (50 µg/mL). Positive clones were confirmed by PCR and sequencing and were subsequently used for protein expression. Primer sequences and PCR protocols are provided in Supplementary Tables S2 and S3. Protein expression was induced by adding isopropyl-β-D-thiogalactoside (IPTG) to a final concentration of 0.5 mM, followed by incubation at 16 °C for 12 h.

Cells were lysed using TieChui *E. coli* lysis buffer and then centrifuged at 4 °C for 30 min at 10,000 rpm to separate the supernatant from the pellet. Crude proteins from both uninduced and induced cells were used for analysis by 12% SDS–PAGE. The His-SUMO-*Pp*Rcys1 present in the supernatant was purified via Ni-column affinity chromatography. The samples were then dialyzed against 1× PBS for 24 h at 4 °C. To completely remove the His-SUMO tag, 1 unit of SUMO protease (purchased from General Biosystems) was added, and the mixture was incubated at 4 °C for 6 h. The Ni column retained the His-SUMO tag, while the unbound fraction containing tag-free r*Pp*RCys1 was collected. These proteins were then analyzed by SDS–PAGE, and their concentrations were determined via a BSA protein assay kit (Beyotime, Shanghai, China) according to the manufacturer’s instructions. The purified protein was lyophilized and stored at −80 °C.

### 2.4. Identification of PpRcys1 by Liquid Chromatography–Mass Spectrometry (LC-MS)

The SDS-PAGE band corresponding to *Pp*Rcys1 and its mutants was excised and transferred into a microcentrifuge tube. A 50% (*v*/*v*) acetonitrile solution was added, and the mixture was agitated overnight at 37 °C to achieve decolorization. Pure acetonitrile was applied to solidify the precipitate, which was then discarded. Proteins were digested with trypsin, followed by incubation in ammonium bicarbonate at 37 °C water bath for 16 h. The resulting digest was transferred to a fresh tube, mixed with an extraction solution consisting of water and anhydrous acetonitrile at a 1:4 ratio, and acidified with 0.5% formic acid. This mixture underwent sonication, centrifugation, and vacuum drying to obtain protein powder. The powder was reconstituted in a sample solution of water and anhydrous acetonitrile (1:49 ratio), acidified with 0.5% formic acid, then vortexed and shaken. Protein samples were analyzed via LC-MS using the TRIPLETOF 5600+ system for identification.

### 2.5. Antimicrobial Activity Assay of rPpRcys1

To determine the minimum inhibitory concentration (MIC) of r*Pp*Rcys1, a modified microtiter plate assay was performed in accordance with Clinical and Laboratory Standards Institute (CLSI) guidelines. Bacterial cultures were grown until they reached an OD_600_ of 0.4 and then diluted to 10^4^ CFU mL^−1^ in Mueller–Hinton broth (MHB) medium (HB6232, Haibo, Qingdao, China). r*Pp*Rcys1 and its mutants were dissolved in 1× PBS. Each well of a 96-well plate received 20 μL of peptide solution and 80 μL of the diluted bacterial suspension. A concentration series (64 μM, 32 μM, 16 μM, 8 μM, 4 μM, 2 μM, and 1 μM) was tested. Ampicillin was used as a positive control, and 1× PBS as the negative control. Plates were incubated at 37 °C for 18 h [29]. Minimum inhibitory concentrations were determined by the absence of color change, as indicated by the resazurin assay OD_560_ and OD_590_ [29]. Each condition was assessed with three biological replicates and three technical replicates to ensure reliability.

Following the MIC assay, bacterial growth curves were constructed by measuring OD_600_ values at MIC and 0.5× MIC concentrations at 0, 4, 8, 12, and 24 h. BSA served as the control protein. The minimum bactericidal concentration (MBC) of r*Pp*Rcys1 was determined using a bacterial viability assay. After 18 h of incubation, 10 μL from each well without visible bacterial growth (OD_600_ ≤ control) was streaked onto Mueller–Hinton agar (MHA, HB0128, Haibo, Qingdao, Shandong, China) plates. These were incubated at 37 °C for 24 h, after which surviving colonies were counted. The minimum bactericidal concentration (MBC) was defined as the lowest peptide concentration that resulted in ≥99.9% killing, indicated by the absence of colony formation. To ensure reliability, three biological replicates and three technical replicates were performed for each condition. Ampicillin was included again as a positive control, while 1× PBS served as the negative control. All experiments were performed in triplicate (three biological and three technical) to replicate data robustness.

### 2.6. Molecular Dynamics (MD) Simulations

To investigate whether *Pp*Rcys1 can disrupt bacterial membranes, molecular dynamics (MD) simulations were performed. A mixed membrane model was constructed using the Membrane Builder module of CHARMM-GUI (https://charmm-gui.org/, accessed on 2 February 2025) [30]. The constructed mixed membrane had a surface area of 12 × 12 nm^2^, with each of the upper and lower layers containing 366 POPE molecules and 122 POPG molecules (POPE:POPG ratio of 3:1) [31,32]. Both the membrane and *Pp*Rcys1 were described via the CHARMM36 force field, whereas TIP3P water molecules represented the solvent [33]. We simulated two systems: *Pp*Rcys1 alone and *Pp*Rcys1 in the membrane. Solvent boxes of 9.34 × 9.34 × 9.34 nm^3^ and 12 × 12 × 16 nm^3^ were constructed for the former and latter systems, respectively. In both systems, Na^+^ and Cl^−^ ions were added to ensure electrical neutrality. All MD simulations were performed via GROMACS version 2023.3 [34], and trajectory visualization was carried out via VMD version 1.9.3 [35].

The cutoff for the short-range part of the Lennard–Jones and Coulomb interactions was set to 1.2 nm. The particle mesh Ewald algorithm [36,37] was employed to compute the long-range part of the Coulomb interaction, with a grid space of 0.16 nm and a spline of order 4. The temperature was maintained at 310 K (above the phase transition temperature of POPG and POPE) using the V-rescale algorithm with a time constant of 1.0 ps [38]. The pressure was maintained at 1 bar via the C-rescale algorithm in a semi-isotropic method [39], with a time constant and compressibility set to 5 ps and 4.5 × 10^−5^ (kJ·mol^−1^·nm^−3^)^−1^, respectively. All bonds were constrained via the LINCS algorithm [40], allowing for a time step of 2 fs. For each system, energy minimization was performed first, followed by a 500 ps pre-equilibration in the NVT and NPT ensembles. Subsequently, 500 ns simulations of *Pp*Rcys1 were performed in aqueous solution and mixed membrane systems.

### 2.7. Microorganism-Binding Assay

To assess the binding affinity of rPpRcys1 to bacterial cells, a Western blot assay was conducted, as described in previous studies [41]. A total of 1 × 10^8^ CFU of microorganisms were placed in a 1.5 mL centrifuge tube and incubated with 200 μL of 5 μM His-SUMO-*Pp*Rcys1 protein at room temperature for 1 h under gentle rotation. Following incubation, the cells were harvested, washed three times with 1× TBS, and resuspended. After centrifugation at 10,000 rpm for 5 min, both the bacterial pellet and the supernatant were subjected to SDS–PAGE. His-SUMO-*Pp*Rcys1 was used as a positive control, and the His-SUMO tag alone served as the negative control. Proteins were subsequently transferred onto a polyvinylidene fluoride (PVDF) membrane, which was then blocked with 5% skim milk in 1× TBST. The membrane was incubated with an HRP-anti-His antibody (1:30,000 dilution, Boyi, Changzhou, Jiangsu, China). Detection was carried out via BeyoECL Plus (Beyotime, Shanghai, China) in accordance with the manufacturer’s instructions.

### 2.8. Binding Assay for Pathogen-Associated Molecular Patterns

A modified enzyme-linked immunosorbent assay (ELISA) was used to evaluate the binding of the r*Pp*Rcys1 to bacterial membrane surface components including lipoteichoic acid (LTA), peptidoglycan (PGN), and lipopolysaccharide (LPS) [41,42]. Each component was dissolved in 1× ELISA coating buffer to a concentration of 100 μg·mL^−1^. A 96-well plate (100 μL/well) of each solution was incubated overnight at 4 °C. The wells were washed three times with 1× PBST (pH 7.4) and then blocked with 100 μL of 5% skim milk in 1× PBST for 2 h at 37 °C. After washing three times with 1× PBST (pH 7.4), His-SUMO-*Pp*Rcys1 was dissolved in 1× PBS (pH 7.4) at a concentration of 10 μM and then added to the wells. For comparison, 10 μM BSA served as a control, and 10 μM His-SUMO tag was used as a negative control. The plate was incubated at 37 °C for 1 h and then washed once with 1× PBST (pH 7.4). An HRP-anti-His antibody (1:5000 diluted in 1× PBST, pH 7.4; Boyi, Changzhou, China) was added to the wells, which were subsequently incubated at 37 °C for 1 h. After incubation, the wells were washed five times with 1× PBST (pH 7.4). One hundred microliters of TMB substrate was added for color development, followed by 200 μL of ELISA stop solution. Absorbance at 450 nm was measured using an ELISA plate reader. This experiment was conducted with three biological replicates and three technical replicates to ensure reproducibility.

### 2.9. Electron Microscopy

The electron microscopy protocol was adapted from previous studies [42]. *S. aureus* and *V. alginolyticus* were cultured to mid-log phase in LB medium and 2216E medium, respectively. Bacterial cells were harvested and resuspended in 1× PBS to a final concentration of 10^6^ CFU/mL. The samples were incubated with r*Pp*Rcys1 at its MBC for 2 h on round coverslips placed in 24-well plates. Following treatment, the cells were fixed in 5% glutaraldehyde (prepared in PBS, pH 7.4) for 10 h at 4 °C and subsequently washed three times with 1× PBS. Bacteria treated with BSA served as the control. Samples were dehydrated through a graded series of ethanol (30%, 50%, 70%, 80%, 90%, and 100%) for 10 min at each step at 4 °C. The cells were then subjected to critical point drying (Hitachi-HCP, Hitachi, Tokyo, Japan), sputter-coated with gold (MC1000, Hitachi, Tokyo, Japan), and examined using scanning electron microscopy (SEM, APREO S, Thermo Fisher Scientific, Eindhoven, The Netherlands).

### 2.10. Membrane Permeability Assay

The membrane-disrupting activity of r*Pp*Rcys1s was evaluated using a modified protocol from Yang et al. [43]. Bacterial suspensions of *S. aureus* and *V. alginolyticus* (1–2 × 10⁷ CFU/mL in PBS, pH 7.4) were treated with ampicillin (1× MIC), r*Pp*Rcys1 (1× MIC), 10% Triton X-100 (positive control), or PBS alone (negative control). Following 4 h of incubation at 37 °C, the samples were filtered through 0.22 μm membranes, and the absorbance of the filtrate at 260 nm (OD_260_) was measured. Membrane permeability percentage was calculated using the following formula:

$$\mathrm{Permeability}\;(\%)=\frac{\mathrm{OD}260\left(\mathrm{sample}\right)}{\mathrm{OD}260\left(\mathrm{Triton}\;X-100\right)}\;\times \;100\%$$

### 2.11. Evaluation of Respiratory Chain Dehydrogenase Activity

The inhibitory effects of r*Pp*Rcys1 on bacterial respiratory chain function were assessed by measuring dehydrogenase activity using a tetrazolium reduction assay [44]. Mid-log phase bacterial cultures (OD_600_ ≈ 0.4) were mixed with equal volumes of PBS (pH 7.4), 0.1 M glucose, 2,3,5-triphenyltetrazolium chloride (TTC, 1 mg/mL), and r*Pp*Rcys1. After 5 h of incubation at 37 °C, the mixture underwent phase separation via n-butanol extraction. The organic phase containing reduced triphenyl-formazan (TF) was collected, and its absorbance at 490 nm (OD_490_) was measured spectrophotometrically. Dehydrogenase activity, proportional to TF production, inversely reflects respiratory chain inhibition, with higher OD_490_ values indicating preserved enzymatic function.

### 2.12. Protease Inhibition Assay

*S. aureus* and *V. alginolyticus* were cultured at 37 °C with shaking at 200 rpm for 12 h, harvested (5000× *g*, 3 min), and washed with PBS (pH 7.4). Cells were lysed via sonication (240 W, 2 s on/4 s off, 20 min on ice) and centrifuged (10,000× *g*, 10 min, 4 °C) to obtain supernatants. Test proteins (MIC and MBC in PBS) were mixed with bacterial protease preparations in a 1:1 volume ratio and incubated at 37 °C for 30 min. Protease activity was measured using commercial kits specific for alkaline and neutral proteases (Mlbio, Shanghai, China), with BSA (16 μM) and PMSF (1 mM) used as negative and positive controls, respectively. The protease activity of BSA-treated samples was normalized to 100%. All assays were conducted with three biological replicates and three technical replicates.

### 2.13. DNA Binding Assay

The *Vibrio parahaemolyticus* virulence gene PirA (GenBank: MH410659.1) was cloned into the pSmartI vector using BamHI and XhoI restriction sites to generate the pSmart-PirA plasmid. Each 20 μL binding reaction contained test proteins (8–64 μM), 400 ng of pSmart-PirA plasmid DNA, and binding buffer (1 mM EDTA, 5% glycerol, 20 mM KCl, 1 mM DTT, 10 mM Tris-HCl (pH 8.0), 50 μg/mL BSA). Reactions were incubated at 37 °C for 1 h and then electrophoresed in a 1% agarose gel with 1× TAE buffer at 120 V. BSA (64 μM) served as a control.

### 2.14. Hemolytic Activity Assay

Fish red blood cells (FRBCs) were washed three times with 0.85% saline at 4 °C and were then resuspended to a final concentration of 4% (*v*/*v*). Test proteins (8–32 μM) were incubated with FRBCs at 37 °C for 1 h. Following incubation, the samples were centrifuged, and the absorbance of the supernatant was measured at 570 nm (OD_570_). Hemolysis (%) was calculated using the following formula:

$$\mathrm{Hemolysis}\;(\%)=\frac{\mathrm{OD}570\left(\mathrm{Triton}\;X-100\right)-\;\mathrm{OD}570\left(\mathrm{PBS}\right)}{\mathrm{OD}570\left(\mathrm{sample}\right)-\;\mathrm{OD}570\left(\mathrm{PBS}\right)}\;\times 100\%$$

Triplicate biological and technical replicates ensured the reliability of the data.

### 2.15. Statistical Analysis

Statistical analyses were performed using GraphPad Prism 10.0 (GraphPad, San Diego, CA, USA). Significance was assessed using one-way analysis of variance (ANOVA), and all data are reported as means ± SD. A *p*-value < 0.05 was considered statistically significant.

## 3. Results

### 3.1. Sequence and Structure Characterization of PpRcys1

The protein 20 file of the *P. pollicipes* genome revealed 27,056 protein sequences [16]. AMPs typically comprise about 100 amino acids [45]. During recombinant expression, the signal peptides (approximately amino acids in length) are generally cleaved [46]. Based on this, a screening threshold to 105 amino acids was used. Among the total sequences, 661 were shorter than 105 amino acids, and 119 uncharacterized sequences were retrieved via NCBI BLAST. Of these, only *Pp*Rcys1 exhibited a cysteine content exceeding 10%. The APD3 tool identified *Pp*Rcys1 as a potential AMP, and WoLF PSORT predicted its localization as extracellular.

**Figure 1:**
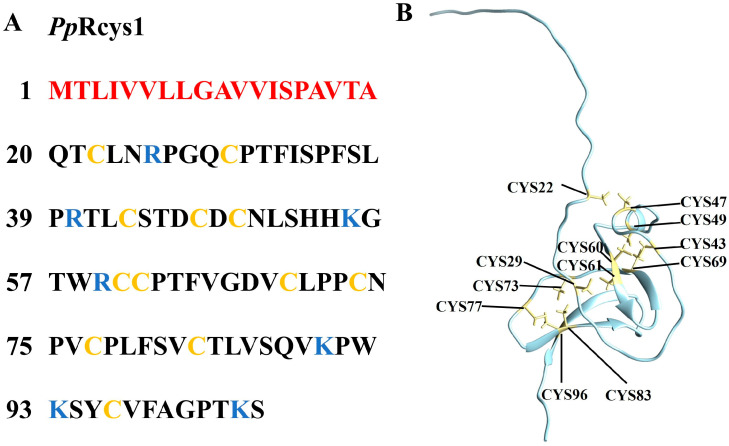
Sequence and structural analysis of *Pp*Rcys1. (**A**) Amino acid sequence of *Pp*Rcys1. Signal peptide residues are shown in red; cationic amino acids (lysine and arginine) are in blue; and cysteine residues are labeled in yellow. (**B**) Predicted 3D structure of *Pp*Rcys1 using AlphaFold2 (https://colab.research.google.com/github/sokrypton/ColabFold/blob/main/AlphaFold2.ipynb, accessed on 2 February 2025). Cysteines are highlighted in yellow.

*Pp*Rcys1 (NCBI accession number: XP_037069689.1) consists of 104 amino acids (Figure 1A), with a predicted molecular weight of 11.18 kDa and a protein isoelectric point of 8.50, as determined by ProtParam. Its net charge is +4.5. The grand average hydropathy (GRAVY) value is 0.48, while Wimley–White whole-residue hydrophobicity is 3.03. Protein-binding potential (Boman index) is 0.32 kcal/mol. The coding sequence (CDS) of *Pp*Rcys1 is 312 bp, with a 255 bp region corresponding to the mature peptide. *Pp*Rcys1 contains a signal peptide at the N-terminus (amino acids 1–19) and 12 cysteine residues, which may form six disulfide bonds (Cys22–Cys47, Cys29–Cys61, Cys43–Cys60, Cys49–Cys69, Cys73–Cys83, and Cys77–Cys96). The AlphaFold2-predicted 3D structure of *Pp*Rcys1 reveals one CSαβ-fold, one β-sheet, and several coils (Figure 1B).

### 3.2. Recombinant Expression and Purification

**Figure 2:**
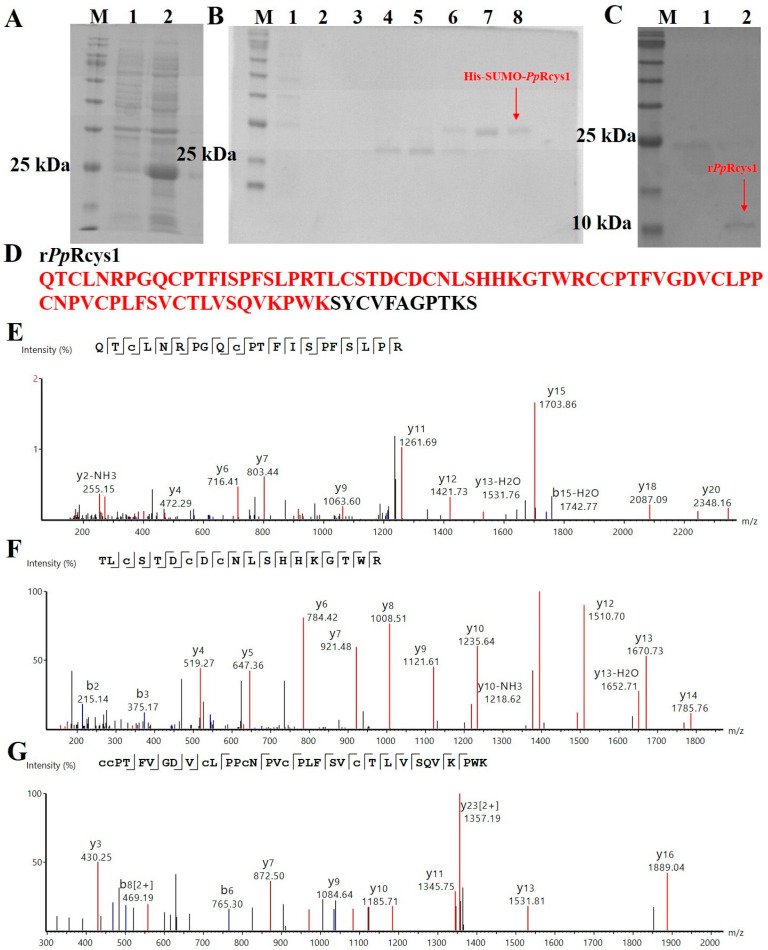
Acquisition process and MS spectrum analysis of r*Pp*Rcys1. (**A**) SDS–PAGE analysis of recombinant *Pp*Rcys1 (r*Pp*Rcys1) expressed with a His-SUMO tag in *E. coli*. Lane M, protein marker; lane 1, total protein obtained from *E. coli* without induction; lane 2, total protein obtained from *E. coli* with IPTG induction. (**B**) His-SUMO-*Pp*Rcys1 was purified via nickel column chromatography. Lane M, protein marker; lane 1, protein not caught by the nickel column; lane 2, equilibration buffer; lane 3, eluent with 20 mM imidazole; lane 4, eluent with 50 mM imidazole; lane 5, eluent with 100 mM imidazole; lane 6, eluent with 150 mM imidazole; lane 7, eluent with 300 mM imidazole; lane 8, eluent with 500 mM imidazole. (**C**) SDS–PAGE analysis of r*Pp*Rcys1 without a SUMO tag. Lane M, protein marker; lane 1, His-SUMO-*Pp*Rcys1 before treatment with the SUMO enzyme; lane 2, tag-free r*Pp*Rcys1; red arrow points to the band of tag-free r*Pp*Rcys1. (**D**) Alignment of mass spectrometry results with the r*Pp*Rcys1 sequence. (**E**–**G**) MS spectra of “ETCVGPGCGPLSSQLVAACENLPAPHPCTFFTCPPGKSCADR”, “TLCSTDCDCNLSHHKGTWR”, and “CCPTFVGDVCLPPCNPVCPLFSVCTLVSQVKPWK”, respectively. The red, blue, and black lines are the y ions, b ions, and noise signals detected by mass spectrometry, respectively.

SUMO tags help mitigate the cytotoxicity of target proteins to host cells and reduce inclusion body formation; they can also be removed enzymatically by SUMO proteases [47]. SDS–PAGE analysis revealed marked differences in the banding patterns of proteins expressed by bacteria, with approximately 25 kDa proteins expressed by the bacteria before and after IPTG induction (Figure 2A). This band corresponds to the expected size of the His-SUMO-*Pp*Rcys1 fusion protein, which consists of a His-SUMO tag (approximately 18 kDa) and a mature peptide of *Pp*Rcys1 (9.36 kDa). His-SUMO-*Pp*Rcys1 can be eluted from the Ni column using a gradient elution with an imidazole eluent. The optimal elution concentration was 500 μM (Figure 2B, lane 7). The yield of His-SUMO-*Pp*Rcys1 obtained from 1 L of *E. coli* culture (OD_600_ ≈ 1.2) was only approximately 100 mg, corresponding to a production level of about 4 μM per liter of bacterial culture. After treatment with the SUMO enzyme, recombinant *Pp*Rcys1 (r*Pp*Rcys1) without the His-SUMO tag was obtained and characterized via SDS–PAGE as approximately 10 kDa (Figure 2C).

### 3.3. Results of LC-MS Identification

LC-MS analysis confirmed the amino acid sequence of r*Pp*Rcys1, identifying three major peptides with overall sequence coverage of 87.06% (Figure 2D–G). These results indicate successful cleavage by SUMO protease during prokaryotic expression, yielding the target protein without residual fusion tags or non-native amino acids. Sequence coverage calculations were restricted to the mature peptide region.

### 3.4. Antimicrobial Activity of rPpRcys1

The antibacterial activity of r*Pp*Rcys1 and its mutants was assessed against two Gram-positive and four Gram-negative bacterial species by determining the minimum inhibitory concentration (MIC) for each. As presented in Table 1, r*Pp*Rcys1 demonstrated notable inhibitory activity against both Gram-positive and Gram-negative bacteria. Specifically, 16 μM r*Pp*Rcys1 effectively inhibited the growth of *V. alginolyticus and E. coli*. The MIC for *A. hydrophila* and *Acinetobacter* sp. L32 was 32 μM. Compared with that of Gram-negative bacteria, the MIC of r*Pp*Rcys1 was lower for *S. aureus* and *Bacillus* sp. T2, both of which were inhibited at a concentration of 8 μM. The minimum bactericidal concentration (MBC) was two- to fourfold greater than the minimum inhibitory concentration (MIC). At a concentration of 0.5 MIC, r*Pp*Rcys1 significantly reduced bacterial growth rates (Supplementary Figure S3).

### 3.5. Results of Molecular Dynamics (MD) Simulations

**Figure 3:**
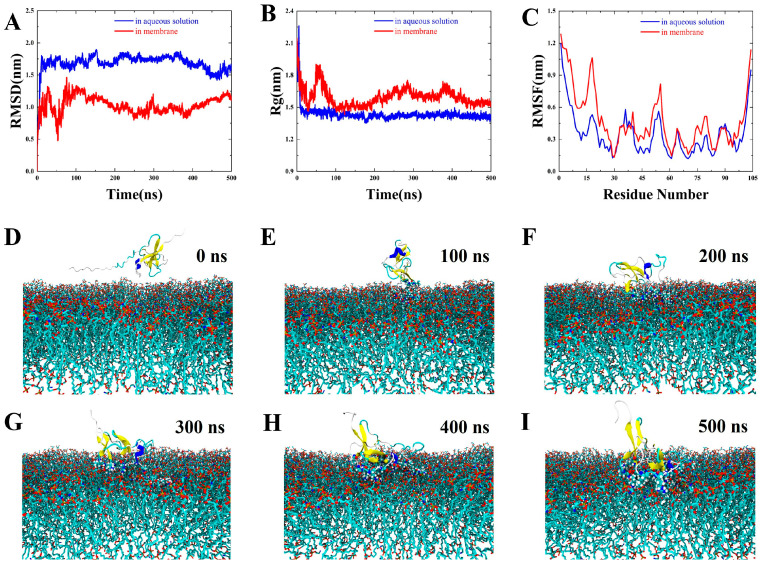
Molecular dynamics simulation results. (**A**) Radius of gyration (Rg) of *Pp*Rcys1 in aqueous solutions and membranes. (**B**) The root mean square distances (RMSDs) of *Pp*Rcys1 in aqueous solution and the membrane. (**C**) Root mean square fluctuations (RMSFs) of *Pp*Rcys1 in aqueous solutions and membranes. (**D**–**I**) Dynamic membrane-binding process of *Pp*Rcys1 during molecular dynamics simulations (0–500 ns); the blue-colored segment represents the Arg40–Lys55 region.

Whether an AMP targets the membrane or intracellular area, initial contact with the bacterial surface is essential. As illustrated in Figure 3, *Pp*Rcys1 spontaneously associates with and embeds into the bacterial membrane. In aqueous solution (blue lines), *Pp*Rcys1 exhibited higher root mean square distance (RMSD) values (Figure 3A) and adopted compact structure (approximately 1.40 nm, Figure 3B), suggesting significant conformational adjustments toward a stable state. In contrast, membrane-bound *Pp*Rcys1 (red lines) showed reduced RMSD fluctuations, indicating restricted conformational changes upon membrane anchoring, with a slightly expanded structure (approximately 1.54 nm after 400 ns) and a dynamically adjusted conformation (Figure 3A). The Arg40–Lys55 region displayed increased root mean square fluctuation (RMSF) values in the membrane-binding state (Figure 3C), suggesting enhanced flexibility potentially critical for membrane interaction (Figure 3D–I).

### 3.6. Microorganism-Binding Activity of rPpRcys1

**Figure 4:**
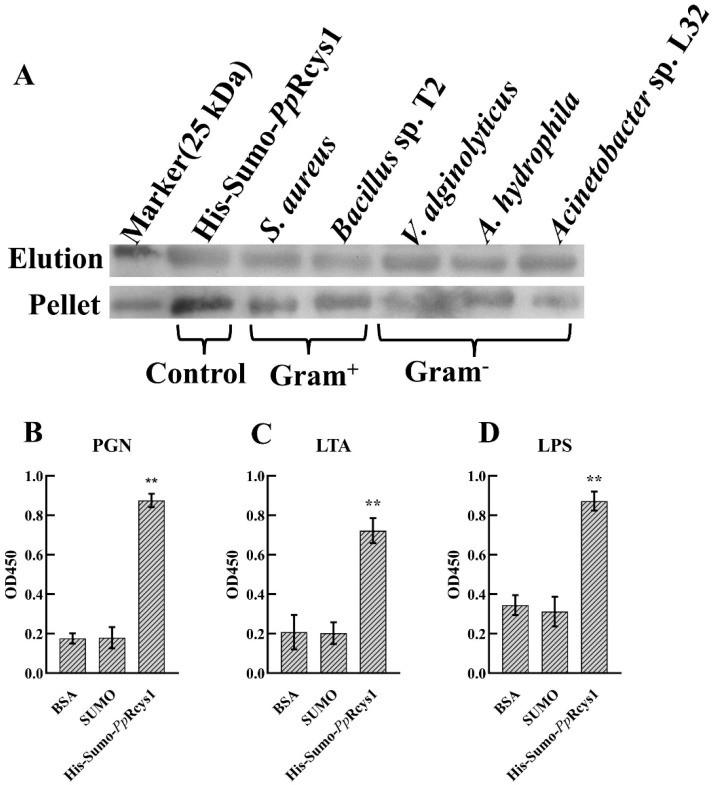
Microorganism-binding activity and PAMP-binding activity of r*Pp*Rcys1. (**A**) Microorganism-binding activity. r*Pp*Rcys1 was detected by Western blot analysis after treatment with bacteria (Gram^+^ and Gram^−^). r*Pp*Rcys1 was used as a positive control. Upper panel, elution fractions; lower panel, final pellet fractions. (**B**) Lipoteichoic acid (LTA) binding activity. (**C**) Peptidoglycan (PGN) binding assay. (**D**) Lipopolysaccharide (LPS) binding assay. r*Pp*Rcys1 was detected via ELISA. r*Pp*Rcys1 was used as a positive control. The SUMO tag was used as a negative control. LTA, lipoteichoic acid; PGN, peptidoglycan; LPS, lipopolysaccharide. The assay included three biological replicates, each with three technical replicates. **, Compared with the control (BSA); *p* value < 0.001.

The His-SUMO-*Pp*Rcys1 fusion protein was used to assess bacterial binding activity. The His-SUMO tag alone (negative control) showed no binding affinity (Supplementary Figure S2). In contrast, r*Pp*Cys1 exhibited strong binding to both Gram-positive bacteria (*S. aureus* and *Bacillus* sp. T2) and Gram-negative bacteria (*A. hydrophila*, *Acinetobacter* sp. L32, and *V. alginolyticus*), suggesting that r*Pp*Rcys1 interact directly with bacterial surfaces (Figure 4A), which may contribute to its antibacterial efficacy.

### 3.7. Pathogen-Associated Molecular Patterns Binding (PAMP) Activity of rPpRcys1

Using His-SUMO-*Pp*Rcys1 for the PAMP-binding assay, the binding affinity of r*Pp*Rcys1 for PAMP (lipoteichoic acid, LTA; peptidoglycan, PGN; and lipopolysaccharide, LPS) was assessed via ELISA. The findings indicated that r*Pp*Rcys1 at a concentration of 10 μM bound to LTA, PGN, and LPS (Figure 4B–D). In contrast, the negative control His-SUMO tag demonstrated no discernible binding to LTA, PGN, or LPS.

### 3.8. Effects of rPpRcys1 on Bacterial Morphology

**Figure 5:**
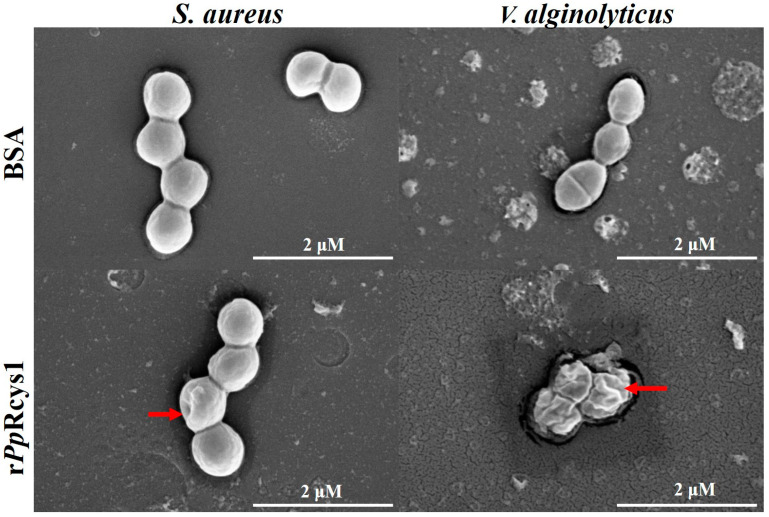
Morphological changes in bacterial cells treated with r*Pp*Rcys1. Morphological changes in bacterial cells treated with r*Pp*Rcys1. Approximately 10^6^ CFU·mL^−1^ bacteria were incubated with the MBC of r*Pp*Rcys1 for 2 h and observed via SEM. PBS was used as a control. The scales are 2 μm. Red arrows indicate deformation in *S. aureus* and membrane wrinkling in *V. alginolyticus*.

Scanning electron microscopy revealed that r*Pp*Rcys1 induced distinct morphological changes in bacterial cells compared to untreated controls. *S. aureus* exhibited abnormal inward deformation, while *V. alginolyticus* displayed pronounced wrinkling of the cell surface (Figure 5), indicating membrane disruption at the MBC (Figure 5).

### 3.9. Effects of rPpRcys1 on Membrane Permeability and the Respiratory Chain

**Figure 6:**
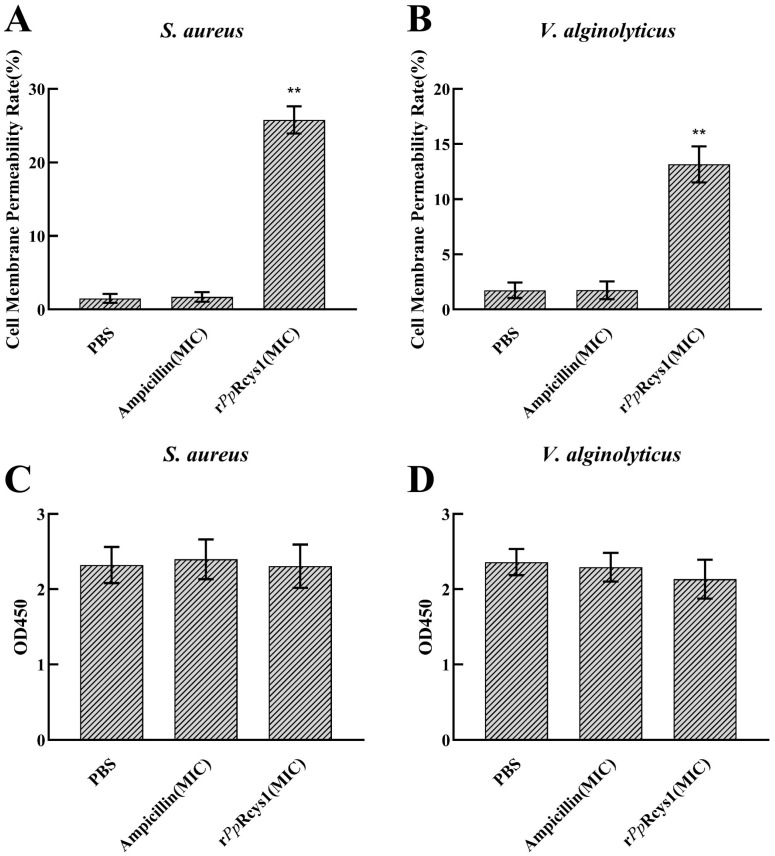
Effect of r*Pp*Rcys1 on membrane permeability and the respiratory chain activity. (**A**) Effect of r*Pp*Rcys1 on the membrane permeability rate of *S. aureus*. (**B**) The impact of r*Pp*Rcys1 on the membrane permeability rate of *V. alginolyticus*. (**C**) Effect of r*Pp*Rcys1 on the respiratory chain dehydrogenase activity of *S. aureus*. (**D**) Effect of r*Pp*Rcys1 on the respiratory chain dehydrogenase activity of *V. alginolyticus.* These assays included three biological replicates, each with three technical replicates. **, Compared with the control (PBS); *p* value < 0.001.

Treatment with recombinant peptide r*Pp*Rcys1 significantly increased membrane permeability in both *S. aureus* and *V. alginolyticus* compared with the PBS control (Figure 6A,B, *p* < 0.001). In contrast, r*Pp*Rcys1 did not significantly affect respiratory chain dehydrogenase activity in either bacterial species (Figure 6C,D, *p* > 0.05). All assays were performed with three biological replicates and three technical replicates.

### 3.10. Effects of rPpRcys1 Protease Activity and DNA Migration

**Figure 7:**
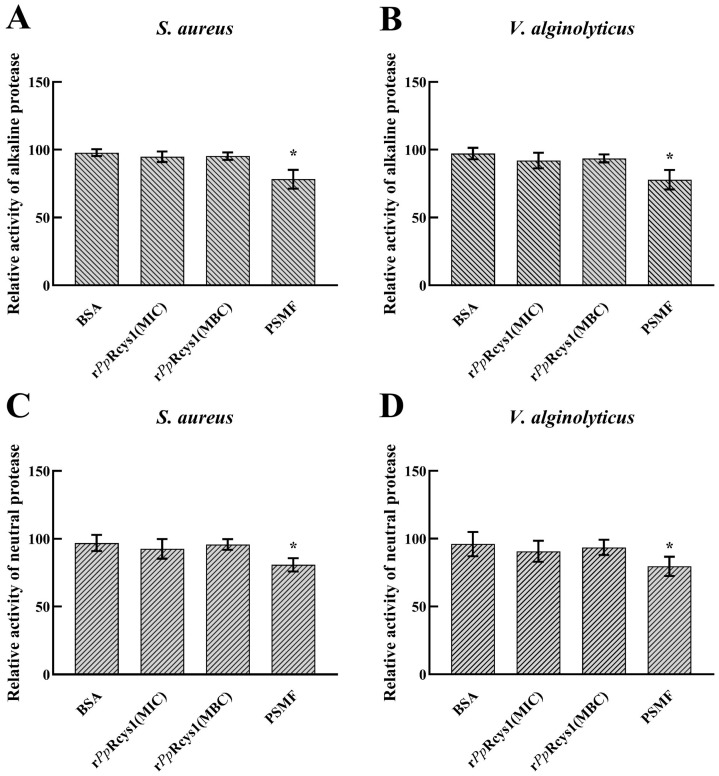
Ability of r*Pp*Rcys1 to inhibit protease activity. (**A**) Ability to inhibit the alkaline protease activity of *S. aureus*. (**B**) Ability of *V. alginolyticus t*o inhibit alkaline protease activity. (**C**) Ability to inhibit the neutral protease activity of *S. aureus*. (**D**) Ability to inhibit the neutral protease activity of *V. alginolyticus*. These assays included three biological replicates, each with three technical replicates. *, Compared with the control (16 μM BSA); *p* value < 0.05.

**Figure 8:**
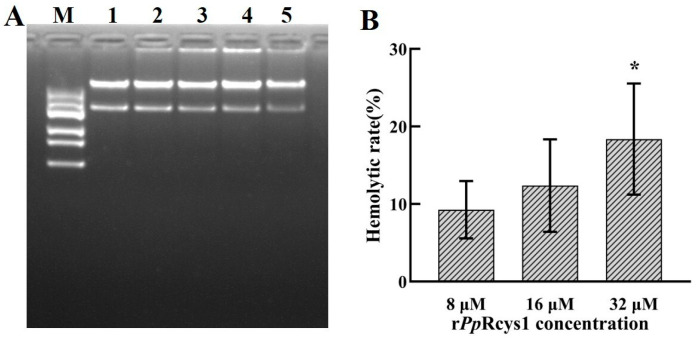
DNA-binding activity and hemolytic activity of r*Pp*Rcys1. (**A**) Binding activity of r*Pp*Rcys1 to plasmid DNA. Lane M, marker; lane 1, 64 μM BSA; lane 2, 8 μM r*Pp*Rcys1; lane 3, 16 μM r*Pp*Rcys1; lane 4, 32 μM r*Pp*Rcys1; lane 5, 64 μM r*Pp*Rcys1. (**B**) Hemolytic activity of r*Pp*Rcys1 on fish red blood cells. The hemolysis rate of the 1× PBS (pH 7.4) treatment was 0%, and that of the 0.2% Triton X-100 treatment was 100%. This assay included three biological replicates, each with three technical replicates. *, Compared with 8 μM r*Pp*Rcys1; *p* value < 0.05.

Relative to the BSA control, r*Pp*Rcys1 treatment did not significantly alter the activities of either neutral proteases or alkaline proteases in *S. aureus* or *V. alginolyticus* (Figure 7A–D, *p* > 0.05). Additionally, there was no detectable difference in plasmid DNA migration rate detected between the r*Pp*Rcys1-treated samples and the control group (Figure 8A, *p* > 0.05).

### 3.11. Hemolytic Activity

Hemolytic activity serves as a key indicator for evaluating the safety of antibacterial drugs. As shown in Figure 8B, the hemolysis rate of fish red blood cells increased with r*Pp*Rcys1 concentration: 9.25% at 8 μM to 12.37% at 16 μM and 18.36% at 32 μM.

## 4. Discussion

AMPs are ubiquitous components of host defense system and exhibit broad-spectrum activity against bacteria, fungi, and viruses, making them highly promising candidates for alternative antimicrobial therapies [48,49]. Given the extraordinary biodiversity and unique ecosystems of marine organisms, marine-derived AMPs have become a focal point in the search for novel bioactive templates [50,51]. Advances in identification and structural characterization methodologies have accelerated the discovery of these marine AMPs and promoted their optimization and synthetic adaptation for therapeutic use [50,52]. Barnacles, which are marine organisms known for their high adaptability and resilience in complex environments, are likely to harbor previously uncharacterized AMPs [17,20]. Therefore, investigating AMPs in *P. pollicipes* represents a valuable avenue for antimicrobial discovery.

Cysteine-rich AMPs are prevalent in various organisms across both plant and animal kingdoms. Examples include cecropins, crustins, and defensins [13,53,54]. These peptides are stabilized by multiple disulfide bridges, which confer resistance to harsh conditions such as salinity and serum proteases, while maintaining antimicrobial efficacy [23]. As a result, cysteine-rich AMPs are considered promising scaffolds for peptide engineering. While existing computational tools can assist in AMP screening from genomic datasets, they often require configuration in Linux environments [55,56]. In contrast, web-based platforms may impose limitations on input size and batch processing [8,57]. Additionally, standard BLAST-based approaches may fail to detect novel AMPs lacking known functional annotations. To overcome these challenges, we developed a Windows-compatible preliminary screening script tailored for cysteine-rich AMPs. This tool complements existing web-based platforms, improving throughput and enabling more efficient AMP identification. The successful discovery of *Pp*Rcys1 validates the efficacy of this approach.

*Pp*Rcys1 exhibits hallmark features of cysteine-rich AMPs: a calculated isoelectric point of 8.5, a CSαβ-fold, and a β-sheet. It was predicted as an AMP in the APD3 database and successfully expressed recombinantly. r*Pp*Rcys1 demonstrated broad-spectrum antibacterial activity against three Gram-positive and four Gram-negative bacteria*. Pp*Rcys1 can inhibit bacteria through a membrane-targeted destruction mechanism. It does not affect the intracellular activities of bacteria, such as protease and respiratory chain enzyme activities or DNA activities. Mechanistically, r*Pp*Rcys1 functions primarily via membrane disruption, rather than through interference with intracellular targets such as proteases, respiratory enzymes, or DNA. These findings suggest that membrane permeabilization is the dominant mode of action for this peptide.

A major challenge in prokaryotic expression of AMPs is their potential cytotoxicity to the host organism, which can impede cell growth and protein yield [47,58,59]. Since antimicrobial activity depends on attaining sufficient peptide concentrations, mitigating toxicity during expression is critical [58,59]. The His-SUMO fusion tag has proven effective in enhancing solubility and reducing toxicity, as shown in prior studies [18,42]. This strategy enabled successful recombinant expression of His-SUMO-*Pp*Rcys1 in *E. coli* BL21(DE3). However, the yield remained relatively low (~100 mg or ~4 μM per liter of culture at OD_600_ ≈ 1.2). Notably, this concentration is below the MIC and MBC values for *E. coli* BL21(DE3), which likely explains the absence of host cell toxicity during protein production.

Several studies have demonstrated that the specific binding between AMPs and bacterial surfaces is a prerequisite for their biological activity [23,60]. Molecular dynamics simulations revealed that *Pp*Rcys1 achieves directional binding with bacterial membranes (Figure 3), a theoretical prediction corroborated by subsequent bacterial binding assays (Figure 4A). Some studies have demonstrated that the specific binding of AMPs to target bacteria serves as a prerequisite for their biological activity [23,60]. Molecular dynamics simulations revealed that *Pp*Rcys1 achieves directional binding with bacterial membranes, a theoretical prediction corroborated by subsequent bacterial binding assays.

The mechanisms by which AMPs disrupt membranes are generally categorized into four primary models: the barrel-stave model, carpet model, toroidal-pore model, and detergent-like model [61]. Among these, the carpet model posits that AMPs accumulate on the membrane surface at high concentrations, disrupt lipid organization, and ultimately induce nonspecific membrane disintegration [62]. Our molecular dynamics simulations revealed that *Pp*Rcys1 binds actively to bacterial membranes and undergoes dynamic conformational changes during this process, destabilizing the lipid bilayer (Figure 3D–I). These characteristics are consistent with the carpet model, suggesting that *Pp*Rcys1 may function through a similar mechanism. Experimental results further confirmed that r*Pp*Rcys1 significantly increases membrane permeability, reinforcing the mechanistic hypothesis generated through simulation.

The antibacterial activity of some peptides arises from synergistic contributions of multiple structures elements [63,64]. RSMF analysis identified the Arg40–Lys55 region of *Pp*Rcys1 as a potentially critical segment for membrane penetration (Figure 3), motivating future site-directed mutagenesis studies. Additionally, the CSαβ-fold demonstrated exceptional structural stability throughout the simulations, likely preserving the peptide’s functional cores during dynamic conformational transitions. This structural robustness may represent a key determinant of *Pp*Rcys1’s potent antimicrobial activity and warrants further validation using mutant variants.

*Pp*Crus-SWD1, a Crustin AMP identified from *P. policipes* [42], also belongs to the cysteine-rich AMP family. Comparative antibacterial assays revealed that *Pp*Rcys1 exhibits superior antimicrobial efficacy. Both peptides appear to operate via membrane-targeted mechanisms consistent with the carpet model, as supported by molecular dynamics data. Notably, *Pp*Rcys1 showed greater structural stability during membrane engagement, which may result from its higher number of disulfide bonds. Furthermore, *Pp*Rcys1 has a lower molecular weight and a higher isoelectric point, features that may enhance electrostatic interactions with negatively charged bacterial membranes. These properties likely contribute to its enhanced antibacterial potency relative to *Pp*Crus-SWD1.

Certain AMPs exploit bacterial surface components such as LTA and PGN as initial binding targets [65,66]. These molecules form a polyanionic matrix that may facilitate AMP translocation across the membranes. The observed ability of *Pp*Rcys1 to bind both LTA and PGN suggests it may utilize a similar entry mechanism, thereby compromising membrane barrier integrity and leading to bacterial death. Scanning electron microscopy confirmed significant morphological disruption, including membrane wrinkling, in r*Pp*Rcys1-treated cells. Consistently, membrane permeability assays demonstrated that r*Pp*Rcys1 compromises bacterial membrane integrity.

While some AMPs exert antimicrobial effects via intracellular accumulation and disruption of vital processes such as DNA replication, protein synthesis inhibition, or enzymatic activity [67,68], our nucleic-acid-binding assays revealed no such capability for *Pp*Rcys1. Unlike AMPs with cationic coiled–coil motifs [68], *Pp*Rcys1’s positively charged residues are embedded within its CSαβ-fold and β-sheet regions, possibly limiting their accessibility. This structural configuration may explain the absence of DNA-binding activity.

Similarly, r*Pp*Rcys1 did not significantly inhibit neutral or alkaline proteases activity in *S*. *aureus* or *V*. *alginolyticus*, nor did it affect respiratory chain dehydrogenase activity. These findings suggest that *Pp*Rcys1 may have a minimal impact on bacterial proteostasis and energy metabolism. However, these null results do not preclude other intracellular targets outside the scope of our current assay. To comprehensively elucidate *Pp*Rcys1’s mechanism of action, future studies should incorporate multi-omics approaches to assess potential intracellular interactions and pathway perturbations.

Lipopolysaccharide (LPS) is a key structural component of Gram-negative bacterial outer membranes. The binding mechanism between AMPs and LPS typically involves an initial electrostatic interaction between the positively charged residues of the AMP and the phosphate groups of the LPS inner core. This is followed by hydrophobic insertion of nonpolar residues—often tryptophan—into the lipid A region [69]. This dual interaction ultimately disrupts the structural integrity of the outer membrane. Our findings suggest that r*Pp*Rcys1 may follow a similar mechanism: the positively charged residues likely facilitate initial electrostatic attraction, while Trp58 and Trp92 may mediate hydrophobic insertion into the lipid A core. This electrostatic–hydrophobic dual mechanism may underlie r*Pp*Rcys1’s potent activity against Gram-negative bacteria.

In addition to its structural role, LPS acts as a virulence factor by triggering inflammatory responses in host organisms [70,71]. Anti-lipopolysaccharide factors (ALFs) are known to neutralize endotoxin activity through direct LPS binding [72,73]. Given the observed binding affinity of r*Pp*Rcys1 LPS, we propose that it may also exhibit anti-endotoxin properties. These hypotheses will be investigated in the future.

Capecchi et al. developed a model for designing non-hemolytic antimicrobial peptides. In this model, peptides that induce less than 20% hemolysis at a concentration of at least 50 μM are classified as non-hemolytic [74]. The hemolysis rate of r*Pp*Rcys1 was 18.36% at a concentration of 32 μM, which is lower than LL-37 and palustrin-OG1, typical antimicrobial AMPs that show over 20% hemolysis at 16 μM [75]. Therefore, r*Pp*Rcys1 can be determined to have relatively low hemolytic toxicity. Leucine, isoleucine, and valine facilitate the insertion of the peptide into the hydrophobic core of the erythrocyte membrane, thereby enhancing its hemolytic activity [76]. In r*Pp*Rcys1, there are seven leucines (comprising 8% of the total amino acids), seven valines (also containing 8%), and only one isoleucine. Consequently, leucine and valine may play a significant role in contributing to the hemolytic activity of r*Pp*Rcys1, which exhibits a hemolytic effect at a concentration of approximately 32 μM.

However, a comprehensive toxicological profile, including cytotoxicity, immunogenicity, and in vivo biocompatibility, remains to be established. These parameters are essential for evaluating the peptide’s potential for therapeutic development and will be addressed in subsequent investigations.

## 5. Conclusions

We established a refined and efficient pipeline incorporating three key criteria—sequence length under 105 amino acids, the presence of a signal peptide, and a cysteine content exceeding 10%—to systematically identify cysteine-rich AMPs from genomic datasets. Using this approach, we successfully identified *Pp*Rcys1 from the genome of the goose barnacle *P. pollicipes*, which demonstrated potent bactericidal activity. Functional assays revealed that *Pp*Rcys1 exerts its antimicrobial effects primarily through targeted membrane disruption, with no detectable interference in intracellular processes such as genomic replication, proteostasis, or respiratory chain enzymatic activity. Future studies will employ multi-omics profiling and functional genomics to further potential intracellular targets and to explore bactericidal mechanisms beyond membrane permeabilization.

## Figures and Tables

**Table 1:** Minimal inhibitory concentrations (MICs) of r*Pp*Rcys1 and its mutants against Gram-positive and Gram-negative bacteria.

Microorganism		Minimal Inhibitory Concentrations (μM)		Minimum Bactericidal Concentration (μM)	
		r*Pp*Rcys1	Ampicillin	r*Pp*Rcys1	Ampicillin
Gram^+^	*S. aureus*	8	2	16	4
	*Bacillus* sp. T2	8	-	32	
	*S. agalactiae*	16	4	32	16
Gram^−^	*A. hydrophila*	32	-	64	-
	*Acinetobacter* sp. L32	32	-	64	-
	*E. coli*	16	64	32	256
	*V. alginolyticus*	16	64	32	-

## Data Availability

All data generated or analyzed during this study are included in this published article, and further inquiries can be directed to the corresponding authors.

## Supplementary Materials

The following supporting information can be downloaded at https://www.mdpi.com/article/10.3390/microorganisms13061381/s1, Figure S1: pSmartI-*Pp*Rcys1 Plasmid map; Figure S2: Original WB figure and Microorganism-binding Assay of SUMO; Figure S3: The inhibitory effect of r*Pp*Rcys1 on bacteria at a concentration of 0.5MIC; Table S1: Encoding sequences of the mature peptides in *Pp*Rcys1 was optimized based on the codon preference of *E. coli*; Table S2: General Primers of pSmartI; Table S3: PCR amplification program. Code S1: Filter procedure code

## Abbreviations

The following abbreviations are used in this manuscript:

AMP	antimicrobial peptide
LTA	lipoteichoic acid
PGN	peptidoglycan
LPS	lipopolysaccharide
Rg	gyration
RMSD	root mean square distances
RMSF	root mean square fluctuations
POPE	1-palmitoyl-2-oleoyl-sn-glycero-3-phosphoethanolamine
POPG	phosphatidylglycerol
