# Supplementary material for: Heterologous Expression and Antimicrobial Mechanism of a Cysteine-Rich Peptide from Barnacle Pollicipes pollicipes
Source: Microorganisms. 2025 Jun 13;13(6):1381. doi: 10.3390/microorganisms13061381 (PMC12195476; doi:10.3390/microorganisms13061381)
Supplement: Supplementary file 1 [file microorganisms-13-01381-s001.zip › supplementary code S1.pdf]

In [3]:
```python
from Bio import SeqIO

def count_fasta_sequences(input_file):
    """
    Counts the number of sequences in a FASTA file.

    Parameters:
    - input_file: Path to the input FASTA file.

    Returns:
    - Number of sequences in the FASTA file.
    """
    count = 0
    for record in SeqIO.parse(input_file, "fasta"):
        count += 1
    return count

def filter_fasta(input_file, output_file, min_length=105):
    """
    Filters sequences from a FASTA file based on the length of the amino acid sequence.

    Parameters:
    - input_file: Path to the input FASTA file.
    - output_file: Path to the output FASTA file.
    - min_length: Minimum length of the amino acid sequence to keep (sequences shorter tha
    """
    with open(input_file, 'r') as infile, open(output_file, 'w') as outfile:
        sequence = ""
        keep = False

        for line in infile:
            if line.startswith(">"):
                # Start of a new sequence header
                if len(sequence) < min_length:
                    # If the previous sequence was short enough, write it to the output fi
                    if sequence:
                        outfile.write(f"{header}\n{sequence}\n")
                # Start collecting the new sequence
                header = line.strip()
                sequence = ""
                keep = True  # Assume we might keep this sequence until we know its lengt
            else:
                # Continue collecting the sequence
                if keep:
                    sequence += line.strip()

        # Check the last sequence collected
        if len(sequence) < min_length and sequence:
            outfile.write(f"{header}\n{sequence}\n")
```

In [2]:
```python
input_fasta = "ejthprotein.fasta"  # Replace with your input file path
```

In [5]:
```python
output_fasta = "Pppeptide_lessthan105.fasta"
```

In [6]:
```python
filter_fasta(input_fasta, output_fasta, min_length=105)
```

In [7]:
```python
num_inputsequences = count_fasta_sequences(input_fasta)
```

In [9]:
```python
num_ouputsequences = count_fasta_sequences(output_fasta)
```

In [8]:
```python
num_inputsequences
```

Out[8]: 27056

In [10]:
```
num_ouputsequences
```

Out[10]: 661

In [24]:
```
def filter_uncharacterized_proteins(input_file, output_file):
    """
    Filters sequences from a FASTA file that contain the "uncharacterized protein"
    string in their description line.

    Parameters:
    - input_file: Path to the input FASTA file.
    - output_file: Path to the output FASTA file where filtered sequences will be written.
    """
    with open(output_file, "w") as out_handle:
        for record in SeqIO.parse(input_file, "fasta"):
            if "uncharacterized protein" in record.description.lower():
                SeqIO.write(record, out_handle, "fasta")
```

In [25]:
```
output_file = "filtered_uncharacterized_proteins.fasta"
```

In [26]:
```
input_file="Pppeptide_lessthan105.fasta"
```

In [27]:
```
filter_uncharacterized_proteins(input_file, output_file)
```

In [28]:
```
num_sequences = count_fasta_sequences(output_fasta1)
print(f"Number of sequences in the FASTA file: {num_sequences}")
```

Number of sequences in the FASTA file: 119

In [29]:
```
from Bio import SeqIO

def filter_high_cysteine_sequences(input_file, output_file, threshold=0.10):
    """
    Filters sequences from a FASTA file where the cysteine ('C') percentage is above the g

    Parameters:
    - input_file: Path to the input FASTA file.
    - output_file: Path to the output FASTA file where filtered sequences will be written.
    - threshold: Cysteine percentage threshold (default is 0.10 or 10%).
    """
    with open(output_file, "w") as out_handle:
        for record in SeqIO.parse(input_file, "fasta"):
            seq_str = str(record.seq)
            cys_count = seq_str.count('C')
            seq_length = len(seq_str)
            if seq_length > 0 and (cys_count / seq_length) > threshold:
                SeqIO.write(record, out_handle, "fasta")
```

In [33]:
```
output_file = "Pp_filtered_high_cysteine_sequences.fasta"
```

In [34]:
```
filter_high_cysteine_sequences(output_fasta, output_file)
```

In [ ]:
```
"""
SignalP 6.0 was employed to predict the presence of signal peptides, while WoLF PSORT was
"""
"""
The physicochemical properties of the protein were predicted using ProtParam.
"""
```
