# Supplementary material for: Heterologous Expression and Antimicrobial Mechanism of a Cysteine-Rich Peptide from Barnacle Pollicipes pollicipes
Source: Microorganisms. 2025 Jun 13;13(6):1381. doi: 10.3390/microorganisms13061381 (PMC12195476; doi:10.3390/microorganisms13061381)
Supplement: Supplementary file 1 [file microorganisms-13-01381-s001.zip › Supplementary.pdf]

Table S1. Encoding sequences of the mature peptides in *Pp*Rcys1 was optimized based on the codon preference of *E. coli*.

Sequences name	Encoding
*Pp*Rcys1	GGATCCCAGACCTGTCTGAATCGCCCGGGTCAG<br>TGCCCGACCTTTATTAGCCCGTTTAGTCTGCCGC<br>GCACCCTGTGTAGCACCGATTGCGATTGCAATC<br>TGAGCCATCATAAAGGCACCTGGCGTTGTTGTC<br>CGACCTTTGTTGGTGACGTGTGTCTGCCGCCGT<br>GCAATCCGGTTTGCCCGCTGTTTAGCGTTTGTAC<br>CCTGGTGAGTCAGGTGAAACCGTGGAAAAGCT<br>ATTGCGTGTTTGCCGGCCCGACCAAAAGTTAAC<br>TCGAG

Table S2. General Primers of pSmartI

Primers	5'-3'
EF	TTA AGA TTC TTG TAC GAC GG
ER	TGC TAG TTA TTG CTC AGC GG

Table S3. PCR amplification program

Program	Temperature	Time	Cycles
Initial denaturation	95 °C	5 min	1
Denaturation	95 °C	30 s	35
Anneal	51 °C	30 s	
Extend	72 °C	1 min	
Final extension	72 °C	5 min	1

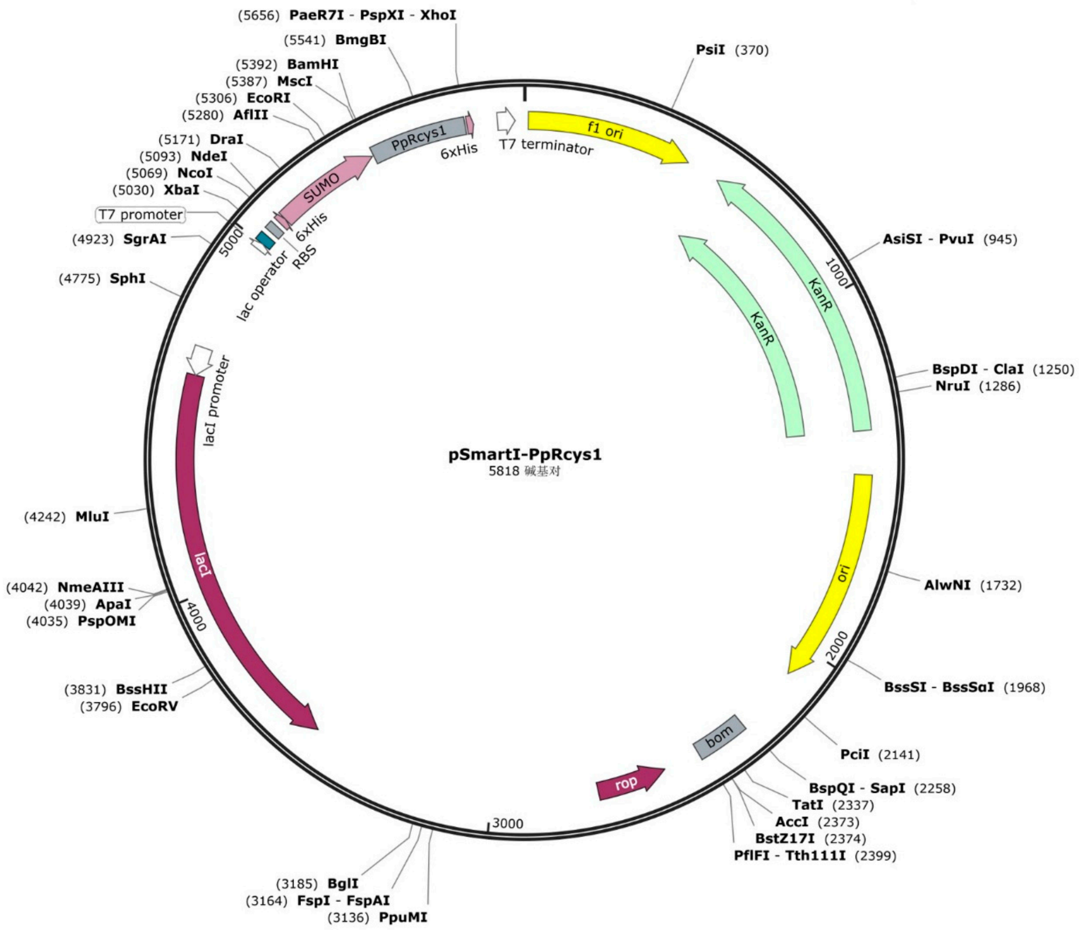

Supplementary Figure S1. pSmartI-*Pp*Rcys1Plasmid map**.**

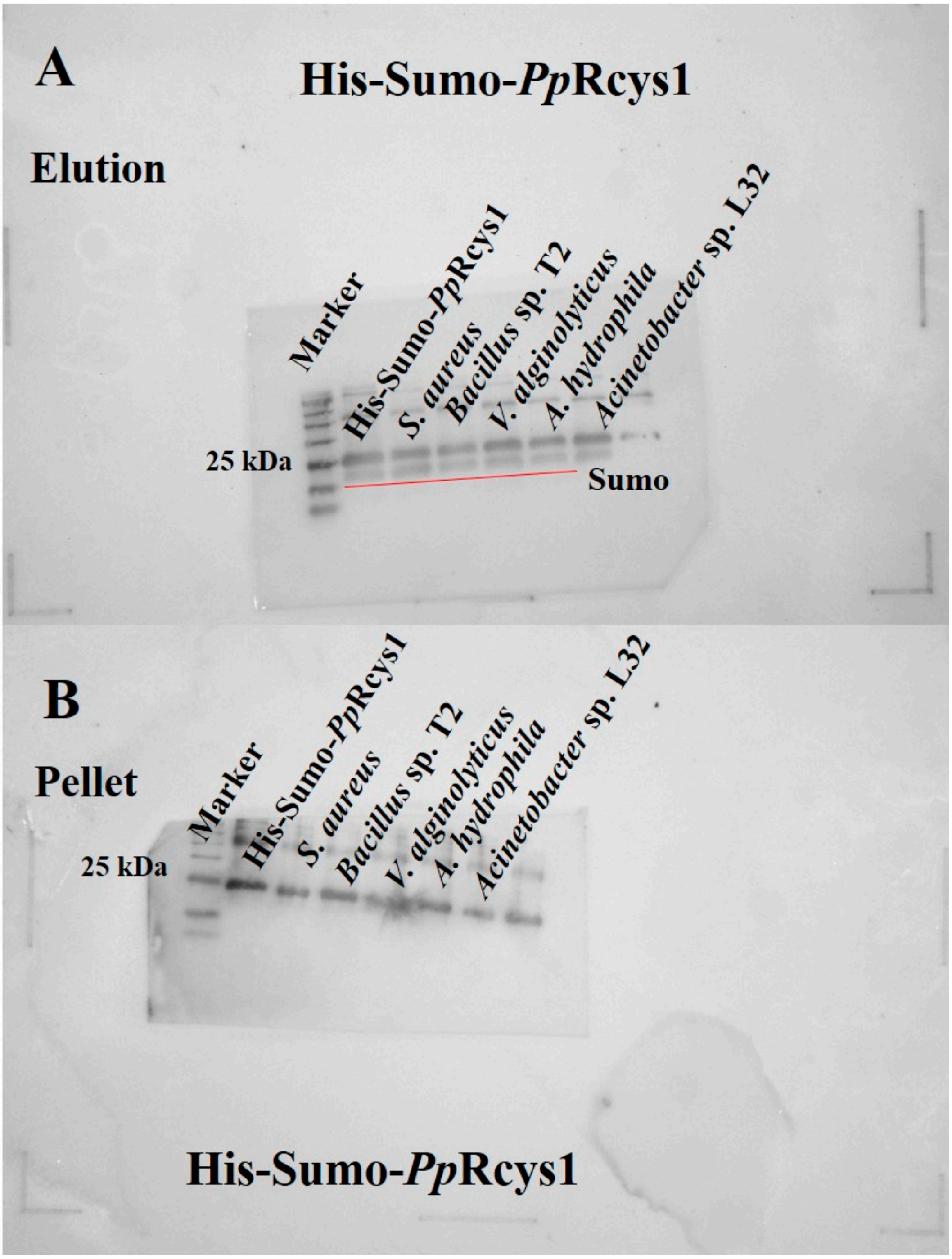

Supplementary Figure S2. Original WB figure and Microorganism-binding Assay of SUMO. (A) Elution fractions of His-Sumo-*Pp*Rcys1. (B) Final pellet fractions of His-Sumo-*Pp*Rcys1.

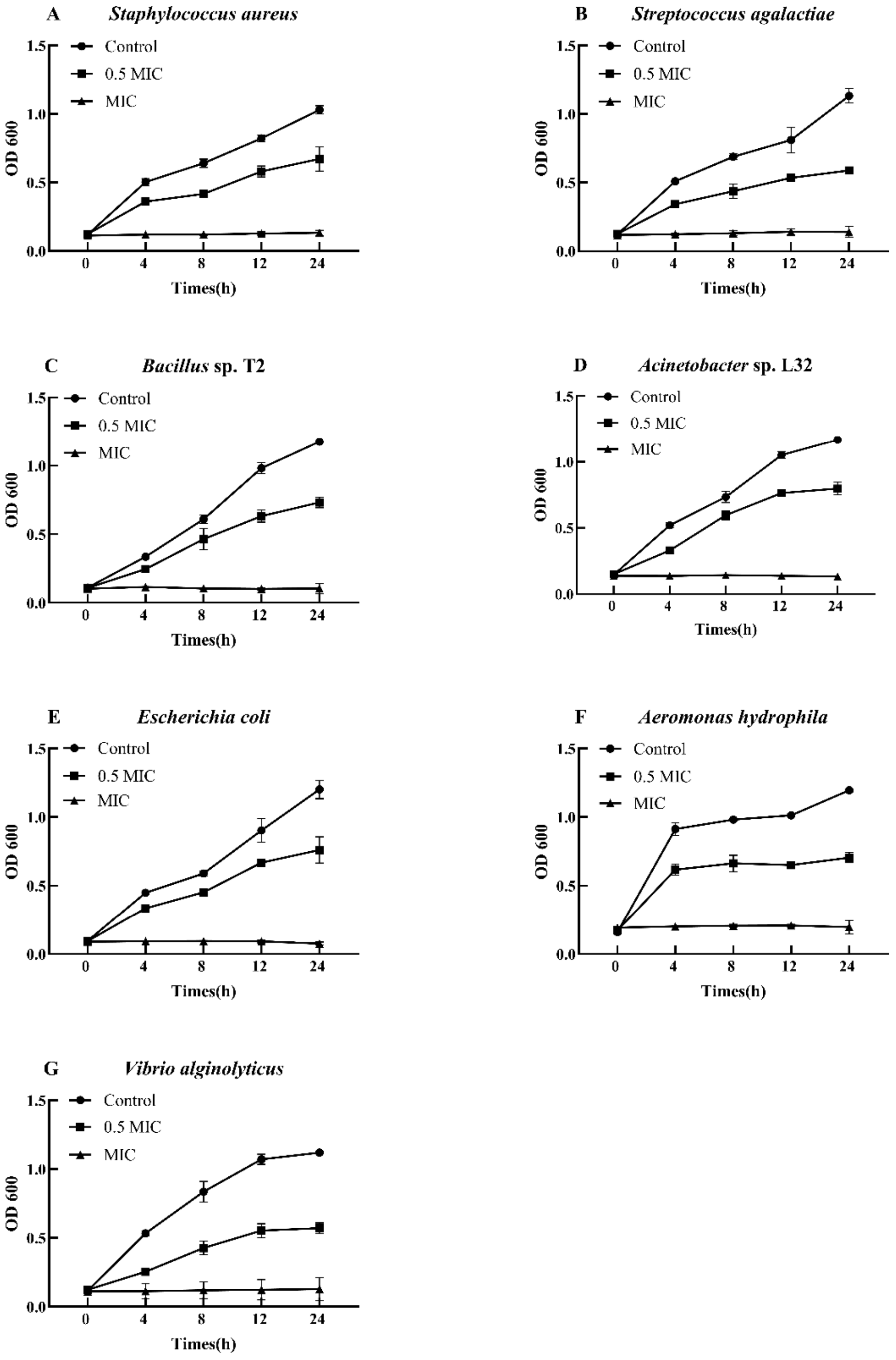

Supplementary Figure S3. The inhibitory effect of r*Pp*Rcys1 on bacteria at a concentration of 0.5MIC.
